# Is personality and its association with energetics sex‐specific in yellow‐necked mice *Apodemus flavicollis*?

**DOI:** 10.1002/ece3.10233

**Published:** 2023-07-04

**Authors:** Beau N. Strijker, Karolina Iwińska, Bram van der Zalm, Karol Zub, Jan S. Boratyński

**Affiliations:** ^1^ Van Hall Larenstein University of Applied Sciences Leeuwarden The Netherlands; ^2^ University of Białystok Doctoral School in Exact and Natural Sciences Białystok Poland; ^3^ Mammal Research Institute Polish Academy of Sciences Białowieża Poland

**Keywords:** allocation, behavior, metabolic rate, performance, personality, sex‐specific

## Abstract

For the last two decades, behavioral physiologists aimed to explain a plausible covariation between energetics and personality, predicted by the “pace‐of‐life syndrome” (POLS) hypothesis. However, the results of these attempts are mixed with no definitive answer as to which of the two most acknowledged models “performance” or “allocation” predicts covariation between consistent among‐individual variation in metabolism and repeatable behavior (animal personality). The general conclusion is that the association between personality and energetics is rather context‐dependent. Life‐history, behavior, and physiology as well as its plausible covariation can be considered a part of sexual dimorphism. However, up to now, only a few studies demonstrated a sex‐specific correlation between metabolism and personality. Therefore, we tested the relationships between physiological and personality traits in a single population of yellow‐necked mice *Apodemus flavicollis* in the context of a plausible between‐sexes difference in this covariation. We hypothesized that the performance model will explain proactive behavior in males and the allocation model will apply to females. Behavioral traits were determined using the latency of risk‐taking and the open field tests, whereas the basal metabolic rates (BMR) was measured using indirect calorimetry. We have found a positive correlation between body mass‐adjusted BMR and repeatable proactive behavior in male mice, which can support the performance model. However, the females were rather consistent mainly in avoidance of risk‐taking that did not correlate with BMR, suggesting essential differences in personality between sexes. Most likely, the lack of convincing association between energetics and personality traits at the population level is caused by a different selection acting on the life histories of males and females. This may only result in weak support for the predictions of the POLS hypothesis when assuming that only a single model explaining the link between physiology and behavior operates in males and females. Thus, there is a need to consider the differences between sexes in behavioral studies to evaluate this hypothesis.

## Abstract 2

The results of the study are in favor of the “performance” model of association between energetics and animal personality, but only in male mice, which represent the boldness/exploration personality. Contrarily, in females, the lack of significant association between their labile behaviors and energetics, combined with opposite correlations with morphological traits compared to males, suggests sex‐specific selective pressure on energy management. Thus, our study supports the suggestion that sex‐specific covariations in pace‐of‐life syndrome exist within the studied population.

## 1 INTRODUCTION

To survive, wild animals need to maintain a balance between acquiring and utilizing energy (Brown et al., 2004; Frappell & Butler, 2004). Even in an inactive state, animals use a significant amount of energy to maintain homeostasis (Speakman, 1997), reflected by standard metabolic rates (SMR) in ectotherms and basal metabolic rates in endotherms (BMR; Burton et al., 2011). The fact that endothermic BMR is several times higher than ectothermic SMR (for a review, see: Bennett & Ruben, 1979; Gillooly et al., 2017) and comprises a significant part of its energy budgets (Portugal et al., 2016; Ricklefs et al., 1996; Speakman, 1999), makes an endothermic animal an appropriate model to study trade‐offs between investments into self‐maintenance and other energetically costly functions. BMR consistently differs among individuals within populations (Auer et al., 2016; Nespolo & Franco, 2007), which is a prerequisite for natural selection to act on this trait (Dohm, 2002). Since BMR was found to be heritable, a significant proportion of the individual variance can be attributed to the additive genetic effect (Nilsson et al., 2009; Wone et al., 2009; Zub et al., 2012). The artificial selection on BMR was found to be effective in experimental studies (Konarzewski et al., 2005). Moreover, BMR undergoes selection with correlated changes detectable at other phenotypic levels, including behavior (Careau et al., 2011; Gębczyński & Konarzewski, 2009).

The consistent among‐individual variation in BMR is considered a significant energetic constraint, shaping the expression of animal behavior (Biro & Stamps, 2010; Careau et al., 2008; Careau & Garland Jr, 2012; Réale et al., 2007). Animal personality refers to among‐individual differences in behavior that persist through time, that is, it is repeatable and a significant proportion of the variance can be attributed to the individual (Biro & Stamps, 2008; Réale et al., 2010; Sih, Bell, & Johnson, 2004). The suites of correlated behaviors (i.e., behavioral syndrome) can be categorized along a continuum ranging from reactive to proactive personality types (Koolhaas et al., 1999; Sih, Bell, Johnson, & Ziemba, 2004). Proactive individuals tend to be more aggressive, explorative, and risk‐taking than reactive individuals, which are less active and more timid. Animal personality traits are heritable and can affect both reproduction and survival (Ariyomo et al., 2013; Brodin & Johansson, 2004; Drent et al., 2003; Haave‐Audet et al., 2022; Moiron et al., 2020; Sih et al., 2012). Consequently, individual variation in behavior is currently a key topic of evolutionary and ecological studies.

The pace‐of‐life syndrome (POLS) hypothesis was originally formulated to explain the variation between populations or species covariances in suites of life‐history and physiological traits shaped by environmental differences (Ricklefs & Wikelski, 2002). Only recently, the assumptions of this hypothesis were extended and it has been proposed that behavior should be included in POLS research, as behavioral traits may play key roles in mediating life‐history trade‐offs (Biro & Stamps, 2008; Réale et al., 2010; Stamps, 2007; Wolf et al., 2007). Moreover, the POLS can also be applied at a within‐population level to explain individual evolutionary strategies being an interplay between energy allocation, acquisition, and mortality trade‐offs mediated by animal behavior (Laskowski et al., 2021). The covariation between physiological and behavioral traits is expected in the evolution of individually distinct life‐history strategies, along a slow‐fast continuum. This groundwork for the POLS hypothesis (Réale et al., 2010) predicts that individual physiological, behavioral, and life‐history traits covary in a population (Careau et al., 2008; Nicolaus et al., 2012; Réale et al., 2000; Schuett et al., 2015).

At least three main conceptual models were developed to predict the relationship between consistent individual variation in metabolism and animal personality within populations (Careau et al., 2008; also see Careau & Garland Jr, 2012; Mathot & Dingemanse, 2015). The “performance” model predicts that proactive individuals, characterized by high levels of activity, exploration, aggressiveness, and boldness, should have a higher BMR than individuals expressing low levels of these behaviors. This model, rooted in the “increased intake” hypothesis, expects a positive association between BMR and energy assimilation. This association allows for the improvement of other physiological functions (Nilsson, 2002) and thus can promote proactive behaviors (Biro & Stamps, 2010; Careau et al., 2008). In contrast, the energy “allocation” model that arises from the “compensatory” hypothesis predicts the opposite, namely a negative relationship between BMR and proactive behaviors (Careau et al., 2008). Here, a high BMR is disadvantageous, at least under limited energy availability and/or challenging environmental conditions, as energetic maintenance competes with investments in other costly functions (Gadgil & Bossert, 1970; Steyermark, 2002). Last but not least, the “independent” model indicates that an increased energy expenditure in activity is not related to maintenance costs, and therefore, BMR does not constrain or promote costly behaviors (Careau & Garland Jr, 2012).

The majority of studies indicate weak but rather positive covariation between behavior and metabolism and thus seem to support the “performance” model (Mathot et al., 2019). However, many other case studies showed either negative or not clear, but context‐dependent, relationships between BMR and behaviors. The main explanation for this discrepancy is likely that most of these studies focused only on the individual level ignoring fundamental variation which defines and separates groups of animals within a population. Likewise, sex‐specific differences are considered an important part of this within‐species variation overlooked in the framework of the POLS (as pointed out by Hämäläinen et al., 2018). Sexual dimorphism, as a result of different selection following distinct reproductive roles and breeding strategies, does not only exist in life‐history optima (Adler & Bonduriansky, 2014; Berger et al., 2014; Bonduriansky et al., 2008; Maklakov & Lummaa, 2013; Mokkonen et al., 2016) and personality (Schuett et al., 2010; Tarka et al., 2018) but also in physiological traits (Lee, 2006; Restif & Amos, 2010; Roved et al., 2016), including BMR (Boratyński et al., 2010, 2016, 2018). A faster life history, higher energy expenditure, and expression of proactive behavior in males, compared with females, are at least expected in polygynous reproductive systems (review by Tarka et al., 2018). This suggests that sexual dimorphism should also be seen in the covariation between traits that leads to the evolution of sex‐specific POLS (Hämäläinen et al., 2018). Although only occasionally studied, the sex‐specific correlation between physiology and personality was found to exist in invertebrates (Moschilla et al., 2019; Videlier et al., 2019, 2021; Yarwood et al., 2021). Also, several studies found a similar phenomenon in vertebrates, for example, rodents (Lantová et al., 2011; Šíchová et al., 2014), birds (Bouwhuis et al., 2014), and fishes (Methling et al., 2020).

**FIGURE 1:**
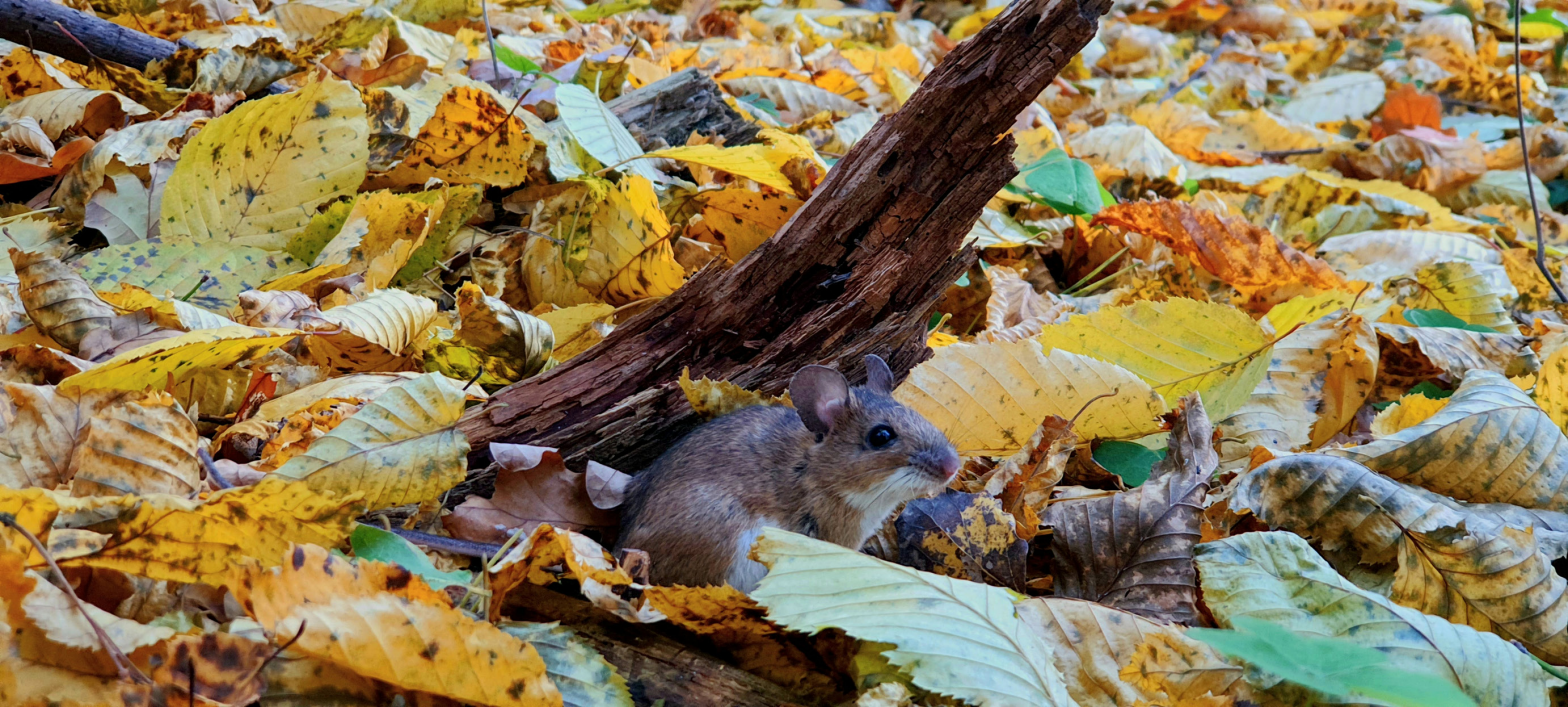
Yellow‐necked mouse after released at study plot (photo by Beau N. Strijker).

Here we aim to test the presence of a sex‐specific relationship between physiology and personality in a single population of yellow‐necked mice *Apodemus flavicollis* (Figure 1), a common small mammal species in temperate forests. In this species, males are more proactive than females (see Bednarz & Zwolak, 2022), thus consistent with the POLS hypothesis, we should also expect sex‐specific differences in metabolism. Yellow‐necked mice represent an example of a polygynous species (Bryja et al., 2008), and thus, sexes should also differ in life histories (Tarka et al., 2018). Under this reproductive system, males are fast‐living and characterized by a predictably shorter life span (under higher predation pressure, see Christe et al., 2006), whereas females (following conclusions by Tarka et al., 2018) should experience lower selection for the association between metabolism and energy expensive behaviors. In turn, in females, predicted slower life histories and investments in future reproduction may result in higher selection for energy allocation. Consequently, if sexual selection acts on the covariation between metabolism and personality, it is expected that the energy “allocation” model applies to females and the “performance” model to males (Hämäläinen et al., 2018). Accordingly, we predicted that BMR will be positively correlated with proactive personality in males but negatively in females.

## 2 MATERIALS AND METHODS

### 2.1 Animals and handling

Animals (in total 37 females and 39 males) were captured on a 1 ha plot in the Białowieża Forest in Eastern Poland during late autumn, between November and December 2021. This study site is mainly formed by deciduous trees, including common hornbeam *Carpinus betulus*, pedunculate oak *Quercus robur*, and small‐leaved lime *Tilia cordata*. The mice were trapped with wooden traps (baited with oat and sunflower seed) and transported to the laboratory of the Mammal Research Institute of Polish Academy of Sciences, located in the Białowieża village, about 10 km away from the trapping plot. The mice were marked with programmable identification transponders (model: IPTT‐300; Biomedic Data Systems Inc.) and kept individually in standard rodent cages (model: 1264; Techniplast). The cages, equipped with a plastic tube as a shelter, were lined with wood shavings. The mice were provided with water, apples, and rodent food (Megan) *ad libitum*. During the implantation of transponders, the mice were anesthetized under a 2% mixture of isoflurane (Iso‐Vet). When the animals were still under anesthesia, we carefully measured their head width (HW) with calipers, as precise as 0.1 mm (Measy DG, Ecotone) and weighed them to the nearest 0.1 g (ScoutPro 200; Ohaus). When all procedures were finished, the mice were released at the exact location of capture. The procedures were repeated in the mice that were recaptured (case of 20 females and 22 males). The repeated measurements were performed on mice that spent around 2 weeks (mean 17 days, Q1–Q3 range = 13–19 days) in natural conditions. The mice were always kept in walk‐in climatic chambers with a temperature of 16 ± 2°C, under a natural photoperiod. The experimental procedures were approved by the Local Committee for Ethics in Animal Research in Olsztyn, Poland (decision no. 67/2020).

### 2.2 Latency and open field tests

On the next day after capture each mouse was exposed subsequently to the latency test (LT) and the open‐field test (OFT), in order to study their behavior. We successfully tested behavior during 116 tests performed for 76 individuals. In total, 41 individuals were tested twice (20 females and 21 males). OFTs were performed during the daytime in an arena (1 × 1 × 1 m) constructed from white polyvinyl chloride plastic. The arena was illuminated with four 4.8 W (470 lm) lightbulbs (Lexman, Leroy Merlin, Białystok) mounted outside the arena above the corners at a height of 1.5 m. Before and after the individual test, the arena was cleaned with 70% ethanol and dried afterward. The digital camera (Hero 5 GoPro Inc.) which allows for making recordings remotely, was mounted at a central point above the arena. The animal was placed in the corner of the arena within the plastic tube from its cage. Each mouse was given 5 min to leave the tube so that we could measure its latency until leaving the known shelter (i.e., LT). Once the animal entered the arena, the tube was immediately removed so it could not affect the OFT. In case the animal did not enter the arena by itself, it was removed from the tube and placed in the corner using a plastic pipe (1 m tall and 15 cm in diameter). Each individual was allowed to explore the arena for 5 min. During the LT and the OFT, observers left the behavioral room to avoid disturbance and were observing and recording the animal with a smartphone, using the GoPro App (GoPro Inc.).

This procedure allowed us to asses different types of behaviors such as latency until leaving the tube (henceforth latency), time spent in different parts of the arena (center, corners, or edges), time spent grooming (henceforth grooming), the number of crossing the center (henceforth crossing center), number of jumps (henceforth jumping), times rearing (henceforth rearing), and standing (considered rearing without support, henceforth standing). The central part of the arena was considered a 50 × 50 cm square located in the center of the arena. The grooming as well as crossing center, jumping, rearing, and standing were obtained manually by a single observer (BNS). The total distance moved during OFT (henceforth distance moved) and the total immobility time was obtained automatically with the open‐source programme ImageJ (Schindelin et al., 2012) with the use of the Animal Tracker plugin (Gulyás et al., 2016).

### 2.3 Respirometry measurements

Metabolic rate was measured 1–2 days after OFT using indirect calorimetry in an open‐flow respirometry system that was designed for measurements of six animals at once. In total, metabolism was measured in 75 individuals (37 females and 38 males) and repeated in 34 mice (18 females and 16 males). For BMR measurements at the thermoneutral zone (~30°C; Cygan, 1985), the animals were placed in custom‐made glass chambers (300 mL) connected to the system with the flow rate through the chambers set to 200 mL/min. Each individual was measured for 3–4 h during daylight. The air was pulled from outside, dried (silica gel 2–5 mm; Chempur), and pushed through an 8‐channel flowmeter (FlowBar‐8; Sable Systems International; henceforth SSI) that allowed for us to set and measure the flow rate separately for each chamber and two baselines. Measurements of oxygen concentrations were done at two lines with two oxygen analyzers (FC10, SSI). Three respirometry chambers were connected on each line and a multiplexer (MUX, SSI) was used to switch between the air stream from the baselines and chambers automatically. The air from each chamber was sampled for 2 min, every 10 min during ~3–4 h session. Thus, during a measurement session, each animal was sampled at least 20 times. The air stream leaving the animal chamber was dried once more and subsampled at a rate of 80 mL/min to pass through each gas analyzer.

### 2.4 Data processing

Scaled mass index (SMI) was calculated following Peig and Green (2009), based on standardized major axis regression between body mass (*m*_b_) and HW. Oxygen consumption (VO_2_) was calculated using equation 10.2 from Lighton (2008), assuming a 0.8 respiratory exchange ratio. MR was calculated as the average of the lowest 30 s of the last stable 60 s reading of each 2 min individual sample of VO_2_. BMR was assumed as the lowest 30 s average for individual mice obtained during a given measurement session.

### 2.5 Statistics

All statistic calculations were made using R 4.1.2 (R Core Team, 2020). *m*_b_, HW, SMI, and BMR were compared between male and female mice using linear mixed effect (LME) modeling procedures, using function “lmer” of package “lme4” (Bates et al., 2015). In the models, sex was included as a centered continuous predictor and animal ID as a random effect. In the LME for BMR, *m*_b_ was included as a covariate.

The repeatability of each behavioral, morphological, and physiological trait was tested separately for sexes using “rptR” package (Stoffel et al., 2017). Since animals can behave differently when naive, the test sequence (first and second time exposed to LT and OFT) was always kept as a fixed factor in the models' for behavioral traits. In each model, animal ID was maintained as a random effect. To estimate the repeatability of BMR, individual *m*_b_ was included as a covariate. In the models for physiological and morphological traits, as well as continuous behavioral traits, the Gaussian distribution of errors was assumed. Behavioral traits, such as crossing center, jumping, rearing, and standing were analyzed against a Poisson distribution and with a square root link function. The latency until leaving the tube showed a binomial distribution (i.e., the animals left the tube in a minute or did not leave till the end of the test). Consequently, we transformed this data to 1 (i.e., left the tube) or 0 (i.e., did not leave the tube). For this reason, latency was analyzed with the assumption of Binomial distribution using logistic regression.

The association between behavioral and physiological or morphological traits was tested in models where both sexes were included. However, since within each sex different behaviors were repeatable (see Section 3; Table 1), we tested the interactions between the sex and the given continuous predictor (HW, residual BMR, and SMI) when analyzing behavior. Residual BMR was obtained from its relationship with *m*_b_ (ordinary last‐squared regression). We only analyzed the covariation between behaviors that indicated repeatability at least in a single sex. Moreover, we focused mainly on behaviors that can clearly indicate anxiety (latency tube), exploration (distance moved), and boldness (crossing center). We had no predictions for the behaviors that do not clearly affect energy expenditure (rearing, standing, and grooming) but we also run models for those (see Appendix Table S1). We ran generalized linear mixed (GLME) modeling procedures, with a Poisson distribution and a square root link function for count data, such as crossing center, rearing and standing using the function glmer of package lme4. For latency, the binomial distribution with the logit link function was used. For continuous data, such as grooming and distance moved, we used LME. In each GLME and LME, the animal ID was used as a random effect, sex as a centered continuous predictor, subsequent behavioral test as a fixed factor and residual BMR, HW, and SMI as covariates. We always included interactions between sex and HW, between sex and residual BMR, and between sex and SMI. Best linear unbiased predictions (BLUP) were calculated as conditional modes for random effect ID obtained from GLMEs and LMEs (with significant effects included, but without interactions) conducted for crossing center and distance moved (as well as for covariates: HW, BMR and SMI) using function “ranef” of package “lme4” (function “se.ranef” of package “arm” was used to estimate standard errors). The Pearson product–moment correlation in package “stats” was used to test the relationship between individual predictions for behavior and physiology/morphology in males and in females.

**TABLE 1:** Repeatability estimates for behavioral, morphological, and physiological traits were obtained in female and male yellow‐necked mice.

Trait	Females	Males	95% CI diff lower bound
	*τ* ± SE	*τ* ± SE	
Probability to leave the tube	0.77 ± 0.27*	0.02 ± 0.14	**0.154**
Time spent in the center	0.18 ± 0.18	0.15 ± 0.16	−0.442
Time spent near walls	0.06 ± 0.16	0.22 ± 0.18	−0.312
Time spent in corners	0.12 ± 0.17	0.20 ± 0.18	−0.405
Number of crossing center	0.29 ± 0.18	0.55 ± 0.15**	−0.199
Number of jumps	0.24 ± 0.18	0.29 ± 0.18	−0.449
Number of rearing	0.41 ± 0.19*	0.34 ± 0.19	−0.457
Number of standing	0.51 ± 0.18**	0.69 ± 0.12***	−0.244
Time spent grooming	0.20 ± 0.18	0.48 ± 0.17**	−0.205
Total distance moved	0.14 ± 0.18	0.47 ± 0.17*	−0.155
Immobility time	0.29 ± 0.19	0.09 ± 0.16	−0.287
Body mass (*m*_b_)	0.93 ± 0.04***	0.94 ± 0.03***	−0.088
Head width	0.94 ± 0.03***	0.87 ± 0.06***	−0.062
Standardized mass index	0.47 ± 0.18*	0.64 ± 0.13***	−0.265
Basal metabolic rate (BMR)	0.81 ± 0.09***	0.80 ± 0.11***	−0.269
*m*_b_‐adjusted BMR	0.73 ± 0.13***	0.52 ± 0.16*	−0.194

*Note*: A lower bound for a 95% confidence interval of differences was calculated based on estimates and standard errors for males and females.

Bold ‐ statistically significant estimates or differences.

*<.05, **<.01, ***<.001.

## 3 RESULTS

After capture males were heavier than females (*F*_1,74_ = 64.63, *p* < .001; females: 28.5 g [95% CI: 26.6–30.3 g], males: 38.9 g [95% CI: 37.1–40.7 g]) and had a wider HW (*F*_1,74_ = 33.55, *p* < .001; females: 15.3 mm [95% CI: 15.1–15.5 mm], males: 16.1 mm [95% CI: 15.9–16.2 mm]). Females (32.6 g [95% CI: 31.5–33.7 g]) had a slightly lower initial SMI than males (34.5 g [95% CI: 33.4–35.5 g]; *F*_1,73_ = 6.01, *p* = .017). There was no interaction between *m*_b_ and sex for BMR (*F*_1,84_ = 0.11, *p* = .740). BMR was positively correlated with *m*_b_ (*β* ± SE = 0.92 ± 0.07; *F*_1,88_ = 189.78, *p* < .001) and *m*_b_‐adjusted BMR did not differ between sexes (*F*_1,75_ = 0.47, *p* = .497).

All morphological and physiological variables were repeatable in male and female mice (Table 1). At least partially different behaviors were found to be repeatable in females and males (Table 1). The latency until leaving the tube was only found to be repeatable in females, whereas only males indicated consistent individual variation in the number of times crossing the center, grooming, and total distance moved. Only repeatability of the number and standing was significantly higher than zero in both sexes (Table 1).

**FIGURE 2:**
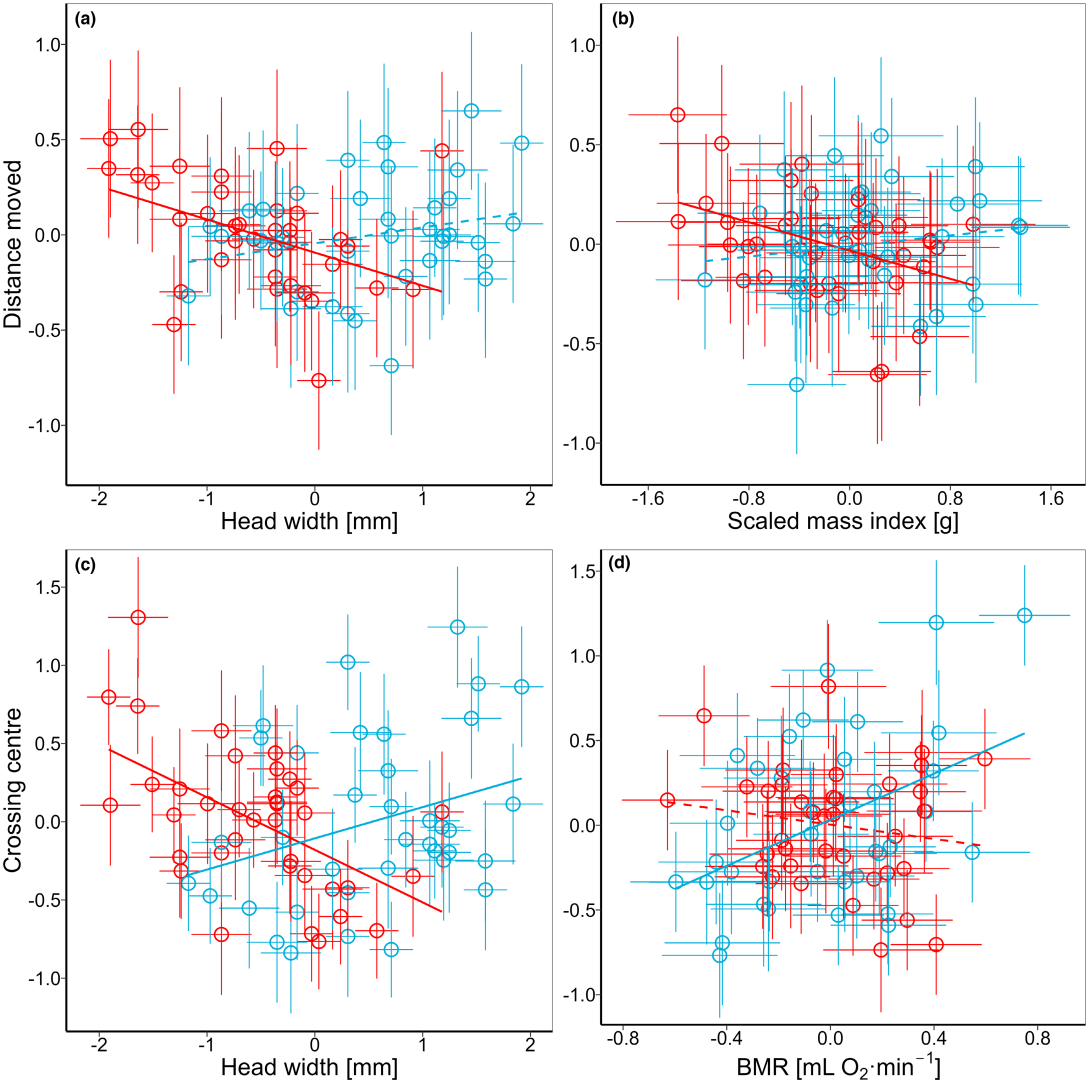
Correlations between best linear unbiased predictions (BLUPs) for (a and b) total distance moved, (c and d) number of times animal crossing center during open field test and the BLUPs for (a and c) head width, (b) scaled mass index, and (d) basal metabolic rate (BMR) in male (blue) and female (red) yellow‐necked mice. Solid lines—significant correlations, dashed lines—insignificant correlations.

There were no significant interactions or general factors explaining the variation in latency until leaving the tube (Table 2). distance moved during OFT decreased between the first (37 m [95% CI: 34–41 m]) and the second session (25 m [95% CI: 21–30 m]). There was no significant interaction between distance moved and residual BMR (Table 2). However, a significant interaction was found between HW and sex for the total distance moved (Table 2). This indicates that total distance moved was negatively related to HW in females (β ± SE = −0.51 ± 0.16, *p* = .004; Pearson *r* = −.43, *p* = .008), but not in males (β ± SE = 0.26 ± 0.16, *p* = .106; Pearson *r* = .24, *p* = .146; Figure 2a). Also, a significant interaction between SMI and sex was found for the distance moved (Table 2), and the SMI was negatively related to the distance moved in females (β ± SE = −0.30 ± 0.13, *p* = .027; Pearson *r* = −.41, *p* = .011) but not in males (β ± SE = 0.18 ± 0.15, *p* = .249; Pearson *r* = .15, *p* = .375; Figure 2b). The number of times that the animal crossed the center decreased significantly between the first (4.6 [95% CI: 3.8–5.5]) and the second measurement session (2.0 [95% CI: 1.4–2.8]; Table 2). The interaction between SMI and sex was insignificant for the explanation of the variation in the number of times crossing the center (Table 2). However, there were significant interactions between HW and sex and between residual BMR and sex for the number of times crossing the center (Table 2). According to the first, the number of times crossing the center decreased with HW in females (*β* ± SE = −0.47 ± 0.15, *p =* .001; Pearson *r* = −.54, *p* < .001), but increased in males (*β* ± SE = 0.42 ± 0.14, *p* = .002; Pearson *r* = .31, *p* = .064; Figure 1c). The second indicates that the number of times crossing the center was positively correlated with *m*_b_–adjusted BMR in males (*β* ± SE = 0.27 ± 0.09, *p* = .004; Pearson *r* = .43, *p* = .007) but was not correlated in females (*β* ± SE = −0.16 ± 0.12, *p* = .117; Pearson *r* = −.16, *p* = .338; Figure 2d).

**TABLE 2:** Results of the models that tested relationships between behaviors indicating anxiety (latency until leaving the tube—latency tube) obtained before the open field test (OFT), exploration (total distance moved by the animal—distance moved), and boldness (number of times animal crossing the center—crossing center) and the following explanatory variables: Residual basal metabolic rates (obtained from ordinary last square regression between metabolism and body mass), head width, scaled mass index, sex (centered continuous predictor), subsequent test, and its interactions.

	Latency tube—anxiety			Distance moved—exploration			Crossing center—boldness		
	es ± SE	*χ* ^2^	*p*	es ± SE	*F*	*p*	es ± SE	*χ* ^2^	*p*
Residual basal metabolic rate	−0.05 ± 0.25	0.03	.853	0.17 ± 0.09	3.48	.066	0.06 ± 0.07	0.69	.405
Head width	−0.49 ± 0.35	2.00	.158	−0.12 ± 0.12	0.95	.333	−0.01 ± 0.10	0.01	.937
Scaled mass index	−0.21 ± 0.30	0.48	.488	−0.06 ± 0.10	0.29	.591	−0.01 ± 0.09	0.01	.912
Sex	1.01 ± 0.71	2.06	.151	0.13 ± 0.23	0.28	.598	−0.05 ± 0.20	0.06	.804
Subsequent test	0.74 ± 0.50	2.23	.135	**−0.85 ± 0.16**	**27.10**	**<.001**	**−0.73 ± 0.12**	**38.76**	**<.001**
Residual basal metabolic rate × Sex	−0.30 ± 0.50	0.35	.552	0.27 ± 0.18	2.17	.145	**0.43 ± 0.15**	**8.32**	**.004**
Head width × Sex	0.32 ± 0.66	0.23	.629	**0.81 ± 0.23**	**11.88**	**<.001**	**0.89 ± 0.20**	**20.49**	**<.001**
Scaled mass index × Sex	−0.61 ± 0.58	1.11	.293	**0.50 ± 0.20**	**5.75**	**.019**	0.19 ± 0.17	1.34	.247

## 4 DISCUSSION

Despite the numerous studies aimed at testing the relationship between personality traits and metabolism (Mathot et al., 2019), such covariations have rarely been analyzed assuming a sex‐specific POLS (Hämäläinen et al., 2018). Nonetheless, few studies indicate that the correlation between personality traits and physiology can differ between sexes in rodents (Lantová et al., 2011; Šíchová et al., 2014), birds (Bouwhuis et al., 2014), fishes (Methling et al., 2020), as well as in invertebrates (Moschilla et al., 2019; Videlier et al., 2019, 2021; Yarwood et al., 2021). This suggests that testing POLS hypotheses and associated trade‐offs should take into account the plausible sexual conflict (Chapman et al., 2003; Wedell et al., 2006), which is associated with different selection acting on male and female life‐histories, energetics, and behavior. According to our predictions, we found a positive correlation between individual variation in *m*_b_‐adjusted BMR and proactive personality in male yellow‐necked mice which can support the “performance” model. However, the lack of consistency in the among‐individual variation in proactive behaviors in females suggests that their phenotypes underlie distinct trade‐offs compared with males. Female mice maintained consistency in the avoidance of risk‐taking (latency until leaving the tube), whereas males did in both boldness (crossing center) and exploration (distance moved). Moreover, at least latency until leaving the tube in females was significantly more repeatable than in males (Table 1), suggesting that the consistency of some behaviors (components of animal personality) differs between sexes. Our study is therefore among the few that support the presence of a sex‐specific correlation, which must be considered when POLS is supposed to be properly tested at inter‐ and intraindividual levels.

As the Darwinian “greater male variability” hypothesis suggests, sexual selection in males is an important evolutionary process that can lead to the tendency toward heterogametic males being more variable than homogametic females, at least in some traits (Zajitschek et al., 2020; see for a current review, in the context of animal personality: Harrison et al., 2021, and important commentary: Del Giudice & Gangestad, 2023). In our study, female and male mice did not show a great difference in any behavior, at least not at the average individual level (but see also Bednarz & Zwolak, 2022). Nevertheless, we found that some behavioral traits were consistent (repeatable) only in one of the sexes, despite using a comparable sample size for both. Females were consistent in latency until leaving the tube (i.e., a safer place) and number of times rearing, whereas males showed repeatability for the number of crossing the center, total time spent grooming, and total distance covered in the arena. Only behavior such as unsupported rearing (i.e., standing) was repeatable in both sexes (Table 1). This indicates that males and females, at least partially, represent distinct personalities. In males, the proactive behaviors (boldness and exploration) can be considered as personalities while in females only the anxiety can. These differences may be associated with the reproductive system of mice and reflect the selection that shapes male and female life–histories. The yellow‐necked mouse has a polygynous mating system (Bryja et al., 2008), where only mothers invest in parental care. This likely resulted in the evolution of sex‐specific life–histories (Brooks & Garratt, 2017), where males and females invest in different fitness components (current and future reproduction). In this kind of reproductive system, characterized by a slower life history, a more reactive behavioral type is expected for females compared with males (Tarka et al., 2018). The fearful behavior of females can allow them to avoid predation, survive the future reproductive event, and successfully raise offspring. Indirect data indicate that in many species of rodents, including yellow‐necked mice, males fall prey to predators more often than females (Christe et al., 2006). This can be attributed to proactive behaviors (i.e., boldness), which can negatively affect survival, even though it promotes reproductive success, at least in males (Smith & Blumstein, 2008; but see also in the context of exploration: Haave‐Audet et al., 2022; Moiron et al., 2020). If this general rule also works in the studied population, the consistent variation in male personality can persist as a result of the evolutionary trade‐off where bold and shy individuals improve different components to realize fitness. Proactive (bold and/or explorative) males, despite being potentially more likely to become prey, can gain fitness benefits since they may fertilize more females. However, if male and female mice differ in life histories and behavioral traits, they may also meet distinct evolutionary constraints between animal personality and physiology (as suggested by Tarka et al., 2018; see also Hämäläinen et al., 2018).

In females, both the number of crossing the center of the arena and the distance that females crossed in the arena were negatively correlated with body size and SMI (Figure 2a–c). It is unclear whether these relationships are age (as suggested in Bednarz & Zwolak, 2022) or body condition dependent (Moran et al., 2021), however, due to a lack of repeatability, they likely have state‐dependent character rather than reflect among‐individual variation (i.e., Godfrey & Bryant, 2000). Contrarywise, consistent among‐individual variation in times crossing center by males (but not distance moved) was positively correlated with body size (Figure 2c). This may suggest that older and/or fast‐growing males represent bolder personalities. Since the number of crossing the center was a repeatable trait in males, the correlation between body size and proactiveness can also be associated with genetic covariation, where the proper physique (e.g., musculature) is needed for behavioral expression (Zablocki‐Thomas et al., 2019). Nonetheless, when corrected for size‐associated variation, crossing the center (but not distance moved) was positively correlated with consistent variation in *m*_b_‐adjusted BMR in male mice only (Figure 2d). Thus, our study supports the "performance" model for males but not for females, suggesting that sex‐specific covariations in POLS exist within the studied population. In support of our results, few studies on distinct animal models indicate the sex‐specific covariation between energy metabolism and behavior. Interestingly, most of them also support the "performance" model in males and/or the "allocation" model in females. The relationship between MR and risk‐taking behavior in Australian field crickets *Teleogryllus oceanicus* was found negative in females, whereas no clear association between all traits was found in males (Moschilla et al., 2019). In the study on ground beetles, *Carabus hortensis* done by Yarwood et al. (2021) both male and female active MR was slightly positively related to exploratory behavior. However, the relationships between active MR and exploration were *m*_b_ dependent in males and temperature dependent in females indicating the different source of this association (Yarwood et al., 2021). Videlier et al. (2019) showed a positive association between SMR and proactivity in male fruit flies *Drosophilla melanogaster*, whereas any association was absent in females. Moreover, after checking for an effect of the activity phase, they found that, in females, there is a significant negative correlation between MR and activity (Videlier et al., 2019). This relationship has a genetic background in males, suggesting that alleles that increase activity also have pleiotropic effects on SMR (Videlier et al., 2021). A positive association between maximum MR, aerobic scope, and boldness and a negative association between those traits and life–span was found in male spotted killifish *Nothobranchius orthonotus*, whereas that trait showed opposite covariation in females (Methling et al., 2020). A study conducted on great tits *Parus major* indicates a negative correlation between BMR and explorative behavior in females and no significant positive in males (Bouwhuis et al., 2014). At least two species of vole indicate the sex‐specific covariation between BMR, however, the direction of the relationship between metabolism and personality was different than in yellow‐necked mice. Male root voles *Microtus oeconomus* and bank voles *Myodes glareolus* indicate rather no relationship between exploration and metabolism, whereas a slightly positive correlation was found in females (Lantová et al., 2011; Šíchová et al., 2014). This indicates that the general trend in the association between metabolism and personality traits is not well understood. However, different associations between behavior and energy metabolism for males and females strongly suggest fundamental differences in the management of their energy budgets (Videlier et al., 2019; this study).

The majority of studies support the “performance” model over the “allocation” model (review in Mathot & Dingemanse, 2015; Mathot et al., 2019) but they do not consider a sex‐specific bias. Although, our results are also in favor of the “performance” model, but only in male mice, which represent the boldness/exploration personality. Contrarily, in females, the lack of significant association between their labile behaviors and energetics, combined with opposite correlations with morphological traits compared to males suggests sex‐specific selection on energy management. Different reproductive roles represented by female and male mice and the evolution of their distinct life‐histories can lead to the antagonistic selection and intralocus sexual conflict (Videlier et al., 2019). The antagonistic selection was reported for both energetics (i.e., Boratyński et al., 2010) and behavior (i.e., Long & Rice, 2007), thus this process may shape the coevolution between those traits. Thus, the males and the females would undergo different models of the association between self‐maintenance and costly behaviors. This, in turn, combined with a predicted distinct association between life–history, physiological, and behavioral traits in males and females (Hämäläinen et al., 2018), may result in a weak effect of energy metabolism on behavior demonstrated at the population level. Thus, the mixed support for the predictions of the POLS hypothesis is among others (see Montiglio et al., 2018; Royauté et al., 2018) very likely originating from the interaction between male and female phenotypes and their unwell understood genetic architectures (Immonen et al., 2018). Therefore, resolving the POLS hypothesis requires accounting for the evolutionary strategies of males and females, plausible intralocus sexual conflict and its consequences on the phenotypic sex‐specific optima (Schenkel et al., 2018).

## AUTHOR CONTRIBUTIONS

**Beau N. Strijker:** Data curation (equal); formal analysis (supporting); investigation (lead); writing – original draft (supporting). **Karolina Iwińska:** Investigation (supporting); writing – review and editing (supporting). **Bram van der Zalm:** Investigation (equal); writing – review and editing (supporting). **Karol Zub:** Investigation (supporting); writing – review and editing (supporting). **Jan S. Boratyński:** Conceptualization (lead); data curation (equal); formal analysis (lead); funding acquisition (lead); investigation (equal); methodology (lead); project administration (lead); resources (lead); supervision (lead); validation (lead); visualization (lead); writing – original draft (lead).

## CONFLICT OF INTEREST STATEMENT

No actual or potential conflicts of interest are declared by the authors.

## Supporting information


Data S1

Table S1

## Data Availability

The complete data set will be accessible through the Appendix Data S1.
