# Supplementary material for: Is personality and its association with energetics sex‐specific in yellow‐necked mice Apodemus flavicollis?
Source: Ecol Evol. 2023 Jul 4;13(7):e10233. doi: 10.1002/ece3.10233 (PMC10318423; doi:10.1002/ece3.10233)
Supplement: Supplementary file 2 — Table S1 [file ECE3-13-e10233-s001.doc]

Table S1. Results of the models that tested relationships between behaviours indicating states as supported (Rearing) and unsupported (Standing) wall-leaning and total time spend grooming (Grooming) and the following explanatory variables: Residual basal metabolic rare (obtained from ordinary last square regression between metabolism and body mass), Head width, Scaled mass index, Sex (centered continuous predictor), Subsequent test, and its interactions.

|  | Rearing | | | Standing | | | Grooming | | |
| --- | --- | --- | --- | --- | --- | --- | --- | --- | --- |
|  | es±SE | *χ*^2^ | *P* | es±SE | F | *P* | es±SE | *χ*^2^ | *P* |
| Residual basal metabolic rate | **0.19±0.10** | **4.04** | **0.045** | -0.15±0.10 | 2.10 | 0.147 | -0.20±0.11 | 3.62 | 0.061 |
| Head width | 0.15±0.16 | 1.07 | 0.301 | **-0.32±0.16** | **3.95** | **0.047** | 0.08±0.14 | 0.31 | 0.582 |
| Scaled mass index | 0.07±0.12 | 0.40 | 0.527 | 0.01±0.12 | 0.01 | 0.908 | 0.14±0.12 | 1.34 | 0.250 |
| Sex | -0.29±0.33 | 0.90 | 0.343 | 0.18±0.33 | 0.31 | 0.575 | -0.04±0.28 | 0.02 | 0.891 |
| Subsequent test | **-0.77±0.13** | **37.51** | **<0.001** | 0.12±0.13 | 0.77 | 0.379 | 0.17±0.19 | 0.79 | 0.378 |
| Residual basal metabolic rate × Sex | -0.09±0.20 | 0.23 | 0.629 | 0.23±0.21 | 1.18 | 0.278 | 0.04±0.21 | 0.04 | 0.849 |
| Head width × Sex | 0.55±0.31 | 3.29 | 0.070 | -0.06±0.31 | 0.04 | 0.852 | -0.25±0.28 | 0.83 | 0.366 |
| Scaled mass index × Sex | **0.59±0.23** | **6.78** | **0.009** | -0.20±0.23 | 0.75 | 0.386 | -0.10±0.24 | 0.17 | 0.670 |
